# A unified workflow for classifying patterns of locoregional failure using radiotherapy treatment planning dose distributions

**DOI:** 10.1093/bjro/tzaf007

**Published:** 2025-05-03

**Authors:** Ceilidh Welsh, Karl Harrison, Sara Lightowlers, Ian Gleeson, Alfred J W Beard, Emma Harris, Gillian C Barnett, Rajesh Jena

**Affiliations:** Department of Oncology, University of Cambridge, Cambridge, Cambridge Biomedical Campus, CB2 0AH, United Kingdom; Cavendish Laboratory, University of Cambridge, Cambridge, CB3 0HE, United Kingdom; Department of Oncology, Cambridge University Hospitals NHS Foundation Trust, Cambridge, CB2 0QQ, United Kingdom; Medical Physics, Cambridge University NHS Foundation Trust, Cambridge, CB2 0QQ, United Kingdom; School of Clinical Medicine, Addenbrookes Hospital, Cambridge, CB2 0QQ, United Kingdom; The Joint Department of Physics, Institute of Cancer Research, London, SW7 3RP, United Kingdom; Department of Oncology, University of Cambridge, Cambridge, Cambridge Biomedical Campus, CB2 0AH, United Kingdom; Department of Oncology, Cambridge University Hospitals NHS Foundation Trust, Cambridge, CB2 0QQ, United Kingdom; Department of Oncology, University of Cambridge, Cambridge, Cambridge Biomedical Campus, CB2 0AH, United Kingdom; Department of Oncology, Cambridge University Hospitals NHS Foundation Trust, Cambridge, CB2 0QQ, United Kingdom

**Keywords:** methodology, local recurrence, radiotherapy, image analysis, image registration

## Abstract

**Objectives:**

This work describes a unified workflow for classifying patterns of locoregional recurrence (LRR) using radiotherapy planning dose distributions. This approach aims to incorporate dose parameters into LRR classifications and facilitate application across different treatment sites and dose prescriptions to standardise classification terminology.

**Methods:**

The relapse diagnostic CT (rCT) and manually delineated relapse gross tumour volume (rGTV) were co-registered with the radiotherapy planning CT (pCT) using deformable image registration (DIR). The DIR accuracy was quantified using the target registration error (TRE) using the absolute centroid distance between cancer site-specific regions of interest (ROIs). Dosimetric structures were delineated for planning regions receiving 95% of the dose prescribed to high-risk, intermediate-risk, and low-risk CTVs, relative to the cancer site or trial. The mapped rGTV was compared relative to each dose structure and classified into one of five categories: central and peripheral high-dose (Type A, Type B), central and peripheral elective-dose (Type C, Type D), and extraneous dose (Type E) failures.

**Results:**

The unified workflow was successfully implemented on two different patient use cases, one from the IMPORT HIGH breast cancer trial, one from the VoxTox head-and-neck study, classifying LRR as Type A and Type E failures, respectively.

**Conclusion:**

This workflow for classifying LRR is applicable across different cancer sites, despite differences in treatment protocol, target dose, and dose delivery. This provides a basis for utilising radiotherapy dose distributions to analyse patterns of failure irrespective of trial design or cancer-site.

**Advances in knowledge:**

Standardised classifications of LRR that are correlated with the planning dose distribution could provide insight into the underlying causes of LRR burden post-radiotherapy and allow for critical evaluation of the current concepts of defined clinical tumour volumes and optimal PTV dose regions.

## Introduction

### Background

Locoregional recurrence (LRR) significantly contributes to cancer-related mortality across major malignancies.^1–3^ Classifying LRRs into distinct categories that describe patterns of failure, and analysing these patterns, could improve our understanding of the treatment factors and biological mechanisms that drive LRR incidence. With advances in radiotherapy, deformable image registration (DIR) has become a valuable tool for investigating recurrence patterns. Currently, DIR workflows are used to spatially map the relapse gross tumour volume (rGTV) from the relapse CT (rCT) to the planning CT (pCT). The rGTV is then classified into one of a series of categories based on its position relative to the original clinical target volume (CTV). The spatial and dosimetric parameters that define these categories for LRR classification have addressed the previous limitations of volume-based terminology used for conventional radiotherapy such as “in-field”, “marginal”, and “out-of-field” that are deemed no longer suitable for heterogenous doses delivered by intensity modulated radiotherapy (IMRT).^4^^,^^5^ This classification approach aims to accurately identify the recurrence site and quantify the dose delivered to it, to analyse failure patterns across a patient cohort. These patterns may reveal associations between LRR and radiotherapy parameters, such as clinical margin definitions, target volumes delineations, and treatment accuracy. Additionally, combining classifications with other omics datasets can investigate broader biological questions relating to LRR, such as the clonal relationship to the original tumour volume.

### Problem statement

The majority of LRR classifications compare the mapped rGTV to clinically defined CTVs for a single cancer site, using geometric and dosimetric criteria to define the classification categories.^6–12^ A CTV-based approach can potentially require redevelopment of the workflow for different cancer sites or trial protocols. This prevents a uniform, consistent approach to classification, with varying definitions and methodologies reducing our ability to collate LRR failure pattern data and draw conclusions. Across current methods, both spatial and dosimetric parameters are typically used to compare the rGTV with multiple site-specific CTVs.^7–10^ These delineated CTVs typically include a high-risk CTV representing the gross tumour volume or bed (dose-level 1) with additional CTVs representing regions at intermediate-risk (dose-level 2) and low-risk (dose-level 3) of microscopic disease. However, the use of site-specific CTVs as the basis for the parameters that determine LRR classifications relies on the assumption that clinically delineated CTVs are an accurate representation of the planned treatment dose distribution. This may not always hold true, especially in the era of IMRT, where treatment planning dose distributions are complex and tailored to multiple dose levels and specific organ-based thresholds. Additionally, clinically delineated CTVs may not be suitable in retrospective trial data with conventional radiotherapy treatment, and cancer-sites where boost dose may cause dose spread outside the CTV, such as boost dose to the pelvis in rectal cancer. Therefore, CTVs could inadequately represent the dose delivered to the failure site, resulting in inaccurate geometric and dosimetric parameters, and a misclassification of the failure.

### Our approach

To address these limitations, we present a workflow that uses the planning dose distribution and clinical dose prescription to determine the spatial and dosimetric parameters for rGTV classification. In this approach, the dose distribution is used to segment regions that receive 95% of the dose prescribed to high-, intermediate-, and low-risk regions as defined by the user for the relevant cancer-site or trial-protocol. Crucially, the 95% dose structures can be consistently segmented across cancer sites utilising the planned dose distribution, eliminating the need for site-specific context from the CTV structures. This facilitates the application of the workflow across cancer sites, as demonstrated in this article using two use cases, one for breast cancer and one for head-and-neck cancer.

### Potential applications

This approach builds upon previous classification methodologies granular classifications and their defined nomenclatures^7^. However, it redefines the spatial and dosimetric parameters using dose-based structures rather than site-specific clinically delineated structures, enabling the development of a unified workflow. This workflow can be applied across different cancer-sites and clinical trials, inspired by the principles of reproducibility, FAIR data practices,^13^ and federated learning. For the specified trial or site, users can provide dose prescriptions and local DIR parameters to the workflow and receive consistent classification terminology that describes LRR failure patterns with respect to the planned dose distribution. This standardised and unified approach is essential for utilising LRR classifications to generate hypothesis and inform clinical trials that aim to investigate recurrence patterns across malignancies.

## Methods

### Patient cohorts: breast and head-and-neck cancer studies

To demonstrate the application of the unified workflow for classifying LRR, one use case is presented for a single breast cancer patient from the IMPORT HIGH trial (08/H0305/13), and one use case for a head-and-neck cancer (HNC) patient from the VoxTox study (13/EE/0008). These individual use cases have been chosen from their cohort to demonstrate how a single unified workflow has been implemented, without redevelopment, across two patient cohorts receiving different radiotherapy approaches (field-in-field IMRT with VMAT simultaneous integrated boost (SIB) and 3D conformal IMRT), with different dose target volumes, anatomical regions of interest (ROIs), and dose prescriptions.

#### IMPORT HIGH: forward planned—field-in-field IMRT

IMPORT HIGH is a phase III, prospective, randomised controlled trial to test whether dose-escalated IMRT after conservative surgery for early breast cancer could reduce radiotherapy side effects, whilst maintaining or increasing cancer cure, in women with higher-than-average local recurrence risk.^14^ Participants were randomised into one of three groups. The control arm delivers standard of care in 23 fractions: with 40 Gy in 15 fractions to the whole breast (WB) plus 16 Gy in 8 fractions sequential photon boost to the tumour bed (TB). The test arms deliver 15 fractions: 36 Gy in 15 fractions to the whole breast; 40 Gy to the partial breast (PB); plus 48 Gy (Test Arm 1) or 53 Gy (Test Arm 2) in 15 fractions simultaneous photon boost to the TB. Patients received follow-up annually for 10 years post-treatment.

#### VoxTox: inverse planned—helical IMRT

VoxTox is a single-centre, non-randomised longitudinal cohort study investigating the relationship between voxel-level radiation dose, and the toxicity experienced by HNC patients following radiotherapy.^15^ Patients were treated as per standard national guidelines, and regional and departmental protocols. Patients undergoing radical radiotherapy received 65 Gy to regions at high-risk of microscopic residual disease (CTV1), 60 Gy to intermediate-risk of microscopic disease (CTV2), and 54 Gy to low-risk regions (CTV3), in 30 fractions. Patients with primary disease of the oral cavity, sinuses, skin, and salivary glands underwent surgical resection with neck dissection where appropriate, with adjuvant radiotherapy at a dose of 60 Gy in 30 fractions. Patients with significant comorbidity received 50 Gy in 20 fractions. Patients received initial post-treatment follow-up at 3 and 6 months after radiotherapy and annually for 5 years post-treatment.

### Deformable image registration

A DIR workflow is performed using Elastix Software^16^ as part of the scikit-rt Python package.^17^ An initial alignment of the pCT and rCT is performed using the centroid of the users chosen site-specific anatomical landmark that is an ROI outlined on both scans. This is followed by a rigid transformation, with affine rotations and linear translations, and finally, a deformable B-spline transform. The final spatial mapping transform that aligns the pCT and rCT is described by the displacement vector field (DVF). This is obtained from the DIR workflow for each patient.

### Registration performance

To evaluate clinically acceptable regions of alignment after DIR both qualitative visual assessment and quantitative assessment is recommended.^18^ In this workflow, quantitative evaluation is assessed using the target registration error (TRE); defined as the residual error between an identified point on the pCT and the transformed rCT. For each patient, the TRE is calculated by determining the absolute distance between the centroid of an ROI delineated on the pCT and the centroid of the equivalent ROI mapped from the rCT to the pCT. This ROI is specified by the user and is chosen due to proximity to the region of LRR instances for that cancer site. The global cohort TRE is defined using the mean of the absolute centroid distance (mm) across all patients from the same cohort evaluated in the DIR workflow. Any patient with a TRE greater than three times the standard deviation of the cohort TRE is deemed an outlier and recommended for exclusion from further analysis due to poor registration performance.

The cohort TRE, which quantifies the registration accuracy, is used as the radius of a synthetic sphere generated around the mapped rGTV centroid. This sphere represents a volume where the rGTV centroid could have been mapped to, based on the registration performance. With a calculated TRE of zero, there would be no spherical volume, and therefore 100% confidence in the registration accuracy and the location of the mapped rGTV centroid. The recurrence sphere is then compared with the delineated dose-based structures to determine the dose delivered to the sphere. This approach ensures that the registration accuracy is incorporated into the final LRR classification.

For DIR registration parameters, individual hospital sites may have pre-existing standardised workflows for assessing registration performance or have a preferred ROI(s) for obtaining the cohort TRE. In this instance, the patient and cohort TREs or chosen ROI(s) can be obtained locally by each research team and provided by the user to the workflow.

### Structures and planning dose distributions

For each cancer site, the prescribed dose levels for each risk region (high, intermediate, low) are provided by the user. The number of relevant risk regions provided will be dependent on the chosen cancer site protocol. For each patient, 95% of the prescribed dose is calculated for each risk region, for example, a 60 Gy prescription to a high-risk tumour volume CTV, results in a 57 Gy 95% dose value used to segment the high-risk dose structure. The 95% dose structure is then delineated using scikit-rt, by creating a new structure mask that includes any voxel in the planning dose distribution that receives ≥ 95% of that prescribed dose, that is, any voxel ≥ 57 Gy. This is repeated for each risk region provided by the user to create a patient-specific dosimetric structure set.

### Classification of failure

**Figure 1.**
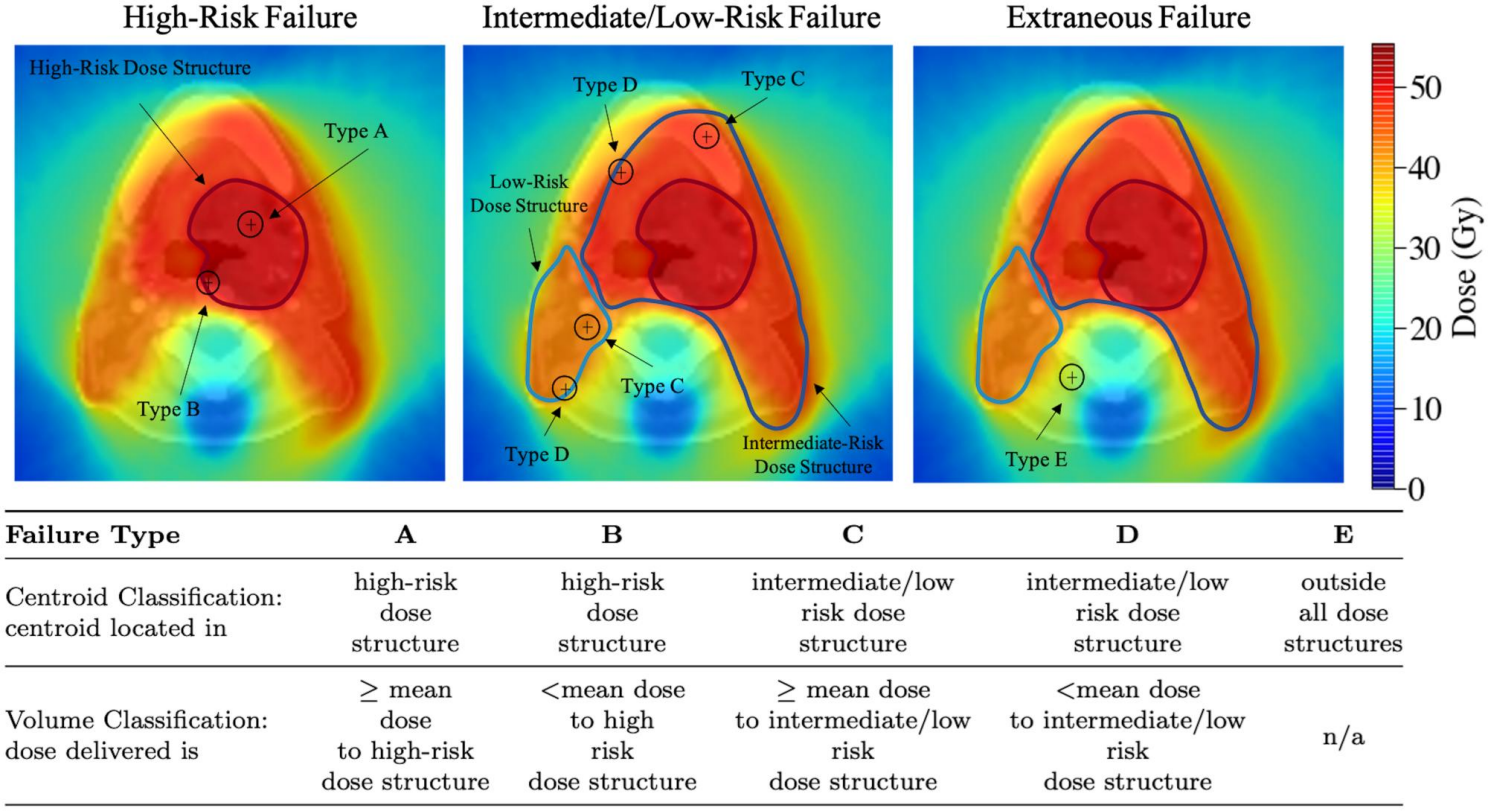
The classification scheme for classifying LRR in head and neck patients based on geometric (centroid) and dosimetric (volume) parameters into categories A-E from high to extraneous dose failures.

Each failure category is defined by two parameters (1) the location of the centroid voxel of the spatially mapped rGTV and (2) the dose delivered to the synthetic recurrence sphere surrounding the rGTV centroid, as illustrated in Figure 1. The centroid voxel and the spherical volume are both compared to the 95% dose structures (high-, intermediate-, and low-risk) to determine the classification category. First, the dose to the rGTV centroid voxel is evaluated and compared to the lowest risk 95% dose structure. If the centroid receives ≥ 95% of the dose value, the centroid is considered within that dose structure. This process is repeated for each dose structure, where the centroid is ultimately classified within the highest dose structure that meets this criterion. This captures whether the centroid lies spatially inside or outside of each 95% dose region. To determine if the rGTV sphere lies outside, inside, or on the boundary of each 95% dose structure requires determining the mean dose to each 95% dose structure. If ≥ 95% of the voxels within rGTV sphere ≥ mean dose in the dose structure, the sphere lies within that dose structure. If the recurrence sphere has < 95% of the rGTV sphere voxels with dose values ≥ mean dose, then the location of the centroid voxel determines whether the sphere is on the boundary of, or entirely outside, the dose structure. Therefore, the final classification combines both the geometric (centroid) and dosimetric (volume) classification into the following categories:

- Type A: central high-dose failure, where the rGTV centroid is within the high-risk dose structure and the dose to ≥ 95% of the rGTV sphere is ≥ mean dose to the high-risk dose structure.
- Type B: peripheral high-dose failure, where the rGTV centroid is within the high-risk 95% dose structure and < 95% of the rGTV sphere is ≥ mean dose to the high-risk dose structure.
- Type C: central elective-dose failure, where the rGTV centroid is within the intermediate-risk 95% dose structure and the dose to ≥ 95% of the rGTV sphere is ≥ mean dose to the intermediate-risk dose structure.
- Type D: peripheral elective-dose failure, where the rGTV centroid is within the intermediate-risk 95% dose structure and < 95% of the rGTV sphere is ≥ mean dose to the intermediate dose structure.
- Type E: extraneous dose failure, where the rGTV centroid is outside both high and intermediate-risk dose structures.

## Results

### Patient examples

The results section demonstrates how the workflow can be applied, without redevelopment, to two distinct use cases. One patient case from the IMPORT breast cancer trial (Patient X) and one from the VoxTox HNC study (Patient Y), to show an example of a high-dose classification (Type A) and an extraneous dose classification (Type E), respectively. To ensure unbiased classification, there was no prior knowledge of the location of the LRR or delineated clinical structures as outlined and reviewed by clinical oncologists.

#### Patient X: IMPORT HIGH

**Figure 2.**
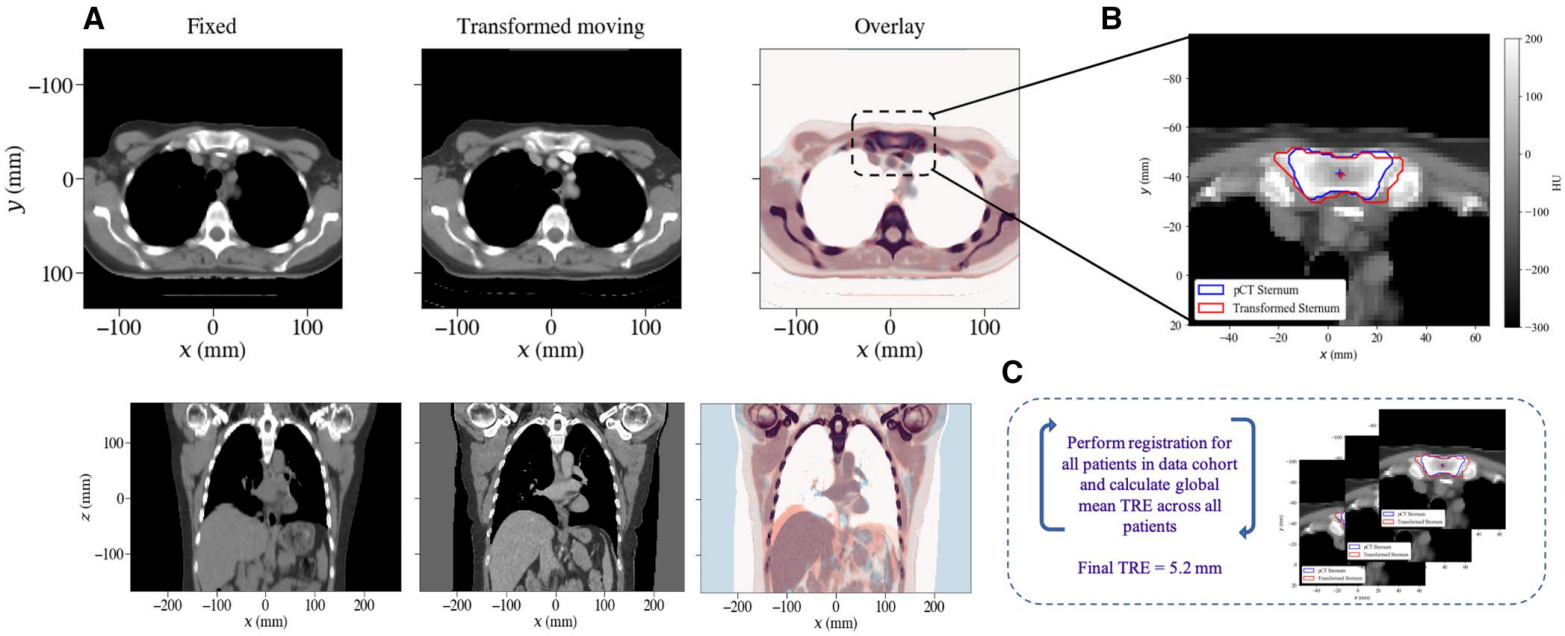
Registration results for Patient X. (A) The fixed image (pCT) and the final result of the transformed moving image (rCT) using B-spline deformation. (B) Overlay of the pCT sternum ROI and spatially transformed rCT sternum ROI. Absolute centroid distance used to calculate individual patient TRE. (C) Perform registration and calculate cohort TRE for all patients.

Patient X had a pCT, with associated clinical planning structure set (WB, PB, TB) and organs at risk (heart, lung, carina, and sternum), and an rCT, with associated relapse structure set, including delineated rGTV and ROIs (carina and sternum). The pCT and rCT were co-registered using the DIR workflow, using the centroid of the sternum (delineated on both scans) as the initial point of alignment (Figure 2), followed by rigid and deformable transformations.

**Figure 3.**
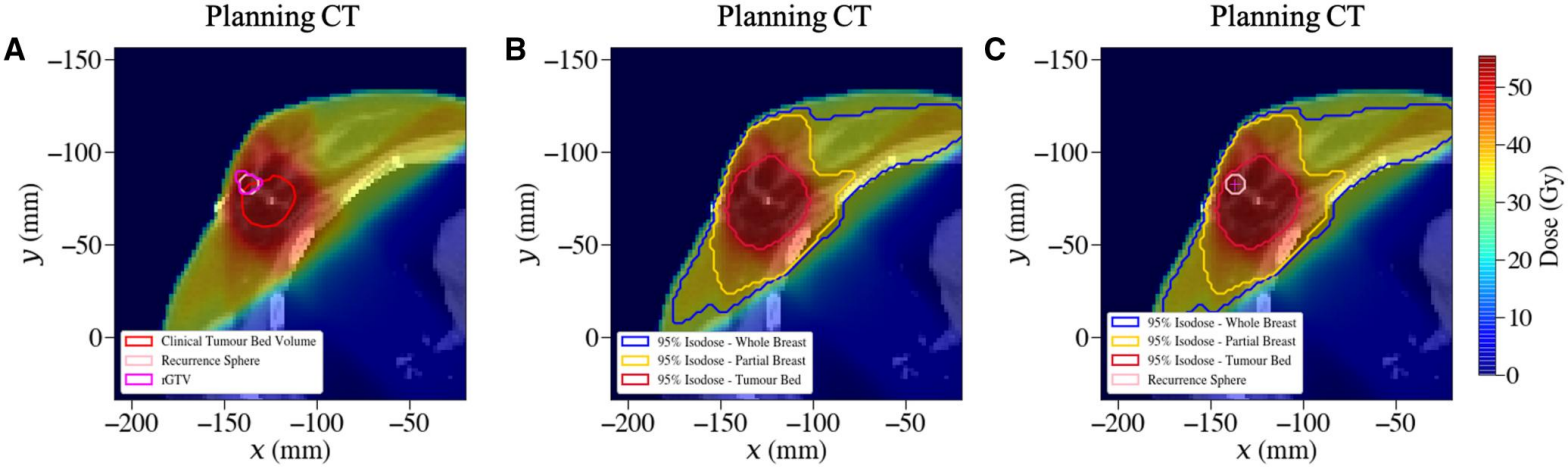
Recurrence structure sets and dose structures for Patient X. (A) Clinical tumour bed volume, mapped rGTV volume as outlined by the radiation oncologist, and the rGTV sphere generated around the mapped rGTV centroid. (B) 95% dose structures generated from the planned treatment dose distribution for 95% dose to high-risk (TB), intermediate-risk (PB) and low-risk (WB) regions to capture dose spread outside clinical CTVs. (C) The planning CT with overlaid 95% dose structures and spatially mapped recurrence sphere and centroid. This demonstrates the difference in classification between a CTV-based comparison (A) and dose structure comparison (C).

The resulting DVF was applied to the rCT structure set, mapping the structures onto the pCT. The final cohort TRE for the IMPORT HIGH dataset was calculated as 5.2 mm. Therefore a spherical volume with a radius of 5.2 mm, equal to the cohort TRE, was generated around the mapped rGTV centroid for Patient X. The clinically prescribed doses for Patient X (randomised into test arm 2 of the trial) were provided by the user for each risk region; 36 Gy to the low-risk WB, 40 Gy to the intermediate-risk PB, and 53 Gy to the high-risk TB. Dose structures were delineated using the planning treatment dose distribution, using 95% of the dose prescribed to each risk region, that is creating structure masks that encapsulate regions receiving 34.2 (WB), 38.0 (PB), 50.4 (TB) Gy, respectively. The mapped rGTV, rGTV sphere and dose structures are visualised in Figure 3A and B, respectively. The mapped rGTV centroid and spherical volume were compared to each dose structure, illustrated in Figure 3C. Both the dose to the centroid (52.8 Gy) and the dose to ≥ 95% of the relapse sphere are greater than the mean dose to the 95% dose structure for the high-risk TB region. Therefore, the LRR was classified as a Type A, high-dose central failure.

#### Patient Y: VoxTox

**Figure 4.**
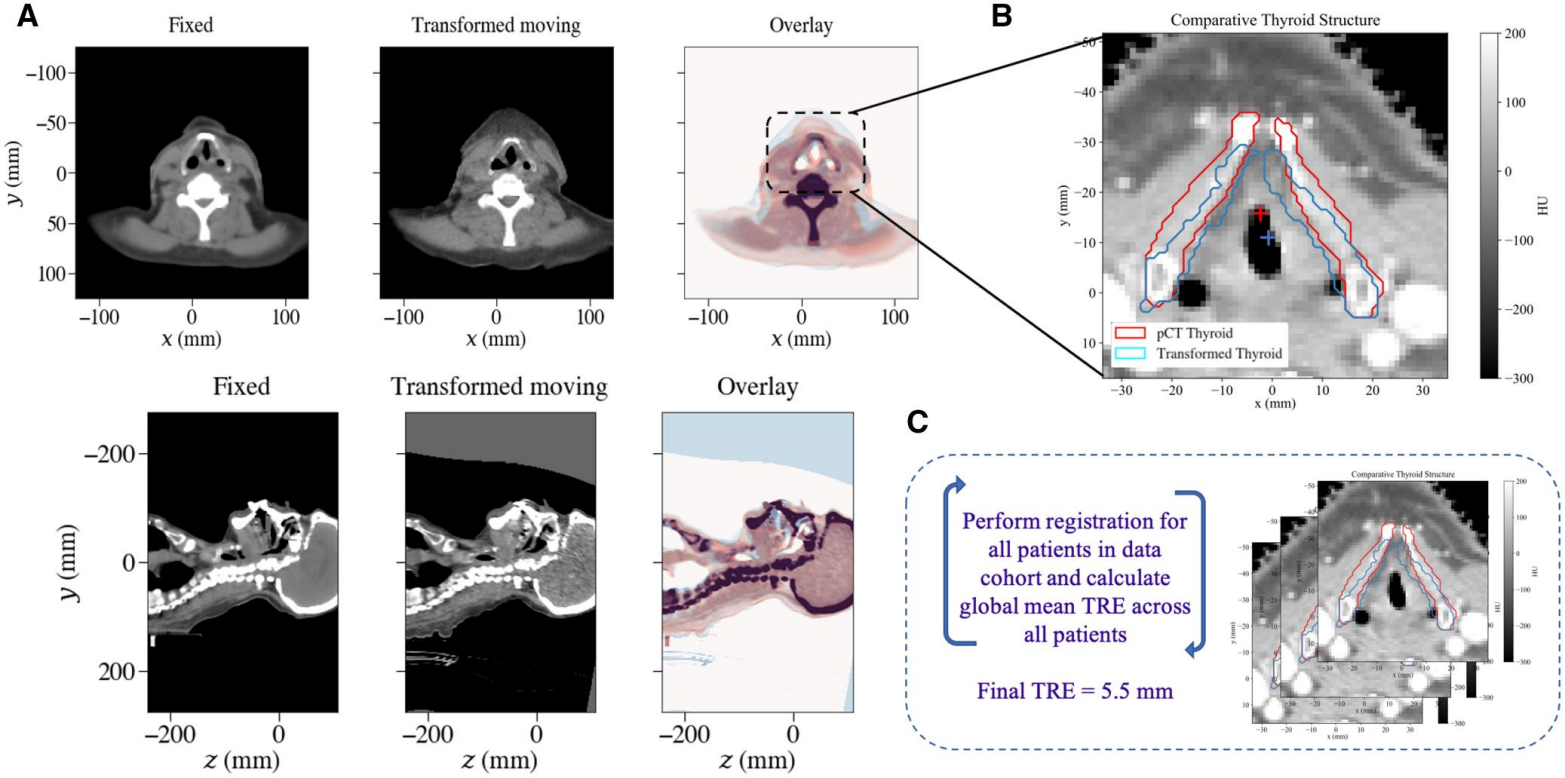
Registration results for Patient Y. (A) The fixed image (pCT) and the final result of the transformed moving image (rCT) using B-spline deformation. (B) Overlay of the pCT thyroid cartilage ROI and spatially transformed rCT thyroid cartilage ROI. Absolute centroid distance used to calculate individual patient TRE. (C) Perform registration and calculate cohort TRE for all patients.

Patient Y had a pCT, with associated clinical planning structure set, including delineated clinical tumour volumes (CTV1) and organs at risk (brain, spinal canal, mandible, eye lens, submandibular glands, parotid glands, cochleas, brain stem, optic nerve, oral cavity, larynx, cricoid cartilage, and thyroid cartilage), and an rCT, with associated relapse structure set, including delineated rGTV and ROIs (cricoid and thyroid cartilage). The pCT and rCT were co-registered using the centroid of the thyroid cartilage (delineated on both CT scans) as the initial point of alignment (Figure 4), followed by rigid and deformable transformations. The resulting DVF was applied to the rCT structure set. The final cohort TRE for VoxTox dataset was calculated as 5.5 mm.

The spherical volume with the cohort TRE radius of 5.5 mm was generated around the sphere of the mapped rGTV centroid. Patient Y received a comorbidity-adjusted protocol, for which there was only a single high-risk CTV and no further risk regions receiving prescribed dose levels. The clinically prescribed dose of 50 Gy was provided by the user.

**Figure 5.**
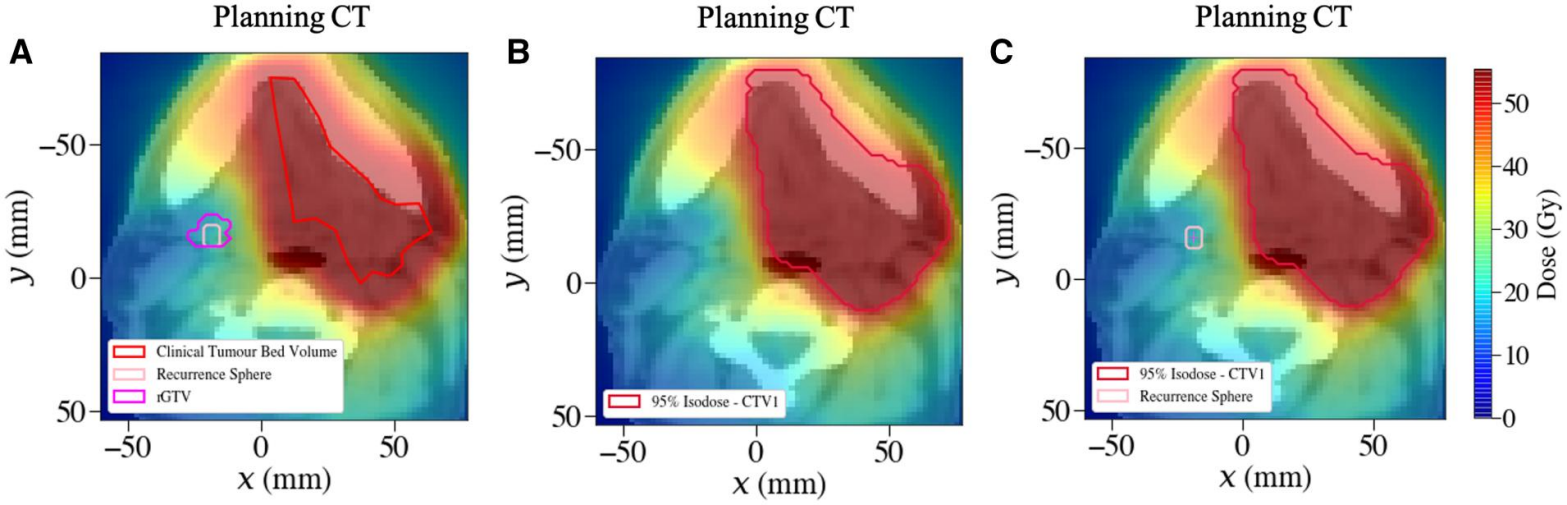
Structure sets for Patient Y. (A) Clinical tumour bed volume, mapped rGTV volume as outlined by the radiation oncologist, and the relapse sphere generated around the mapped rGTV centroid. (B) 95% dose structures generated from the planned treatment dose distribution for 95% dose to high-risk region (CTV1) to capture dose spread outside the clinical CTV. (C) The planning CT with overlaid 95% dose structure and spatially mapped recurrence sphere and centroid.

Therefore, a single dose structure was delineated using the planning treatment dose distribution to capture 95% of the dose prescribed to the high-risk CTV (47.5 Gy). The mapped rGTV, rGTV sphere and dose structure are shown in Figure 5A and B, respectively.

The mapped rGTV centroid and spherical volume were compared to the dose structure, as illustrated in Figure 5C. Both the dose to the centroid (18.3 Gy) and relapse sphere were less than the mean dose to the 95% dose structure for the high-risk TB region. Therefore, the LRR was classified as a Type E, extraneous dose failure.

## Discussion

This article presents a unified classification workflow for LRR across two cancer sites with different radiotherapy protocols: breast cancer, treated with field-in-field IMRT and VMAT SIB, and HNC, treated with 3D conformal IMRT. One patient from each study is presented as a use case to illustrate the generalisability of this workflow in a research setting, and therefore does not imply clinical findings.

This workflow builds on previous work by using spatial and dosimetric parameters to classify LRR failure patterns, but determines these parameters relative to dose-based structures receiving 95% of the prescribed dose for high-, intermediate-, and low-risk regions. Prior methods have defined these parameters relative to CTV structures, which assumes that CTVs are a proxy for delivered dose and the result of dose optimisation and treatment planning is conformal to the clinically delineated volumes. This is not always the case and could result in misclassification, particularly for boost doses or conventional radiotherapy. Therefore, this classification workflow uses dose-based structures as the basis for classification. In radiotherapy dose planning, a 95% isodose must cover the delineated planning target volume (PTV)—the CTV structure plus a site-specific margin—to ensure the CTV receives the clinically prescribed dose. The PTV accounts for differences in planned and delivered dose due to uncertainties in beam alignment, patient positioning, and organ motion or deformation. Therefore, using the 95% dose structures incorporates the dose spread outside of CTVs into LRR classification, representing a more accurate depiction of dose while retaining clinical significance. This change can impact how LRRs are classified. For example, a LRR on the margin of the high-risk tumour CTV would have been spatially defined as a “marginal failure” using purely anatomical methodologies or “Type B” or failure for spatial and dosimetric CTV classifications. However, using 95% dose structures reclassifies certain marginal failures as high-dose failures, more accurately reflecting the dose delivered to these failure sites. An example of this reclassification is demonstrated in the breast cancer use case, illustrated in Figure 3. Aligning classification parameters with dose allows for analysis of radiotherapy treatment effectiveness and LRR patterns, helping to reveal potential associations with radioresistance and treatment parameters that traditional CTV-based classifications might obscure.

Furthermore, the dose-based structures improve the workflows generalisability by utilising the fact that CTV intent is embedded into planned dose distributions, eliminating the need for specifying site-specific CTVs. This streamlines workflow adaptation across cancer sites and protocols, facilitating retrospective analyses and prospective applications with minimal redevelopment, reducing resource requirements, and enhancing cross-institution usability. Additionally, the workflow design could be further adapted for integration with batch-processing and job management systems for research on large cohort studies.

Therefore, we present this workflow as a generalisable approach to LRR classification using dose distributions to improve standardisation of classification terminology and facilitate investigation of LRR failure patterns across different cancer sites or trials. However, we acknowledge there are assumptions and limitations within the workflow. For example, while the 95% dose structures are akin to the PTV, and therefore account for treatment uncertainties, they are not exact representations of the delivered dose to the patient. The use cases presented use planned dose data to demonstrate the generalisability and adaptability of a dose-based classification workflow for research. However, this could be improved further by the incorporation of delivered dose. This is where the adaptability of the methodology can be utilised, and the user can provide delivered dose as the input dose distribution in place of planned dose for trials where this data is collected and available for analysis.

Furthermore, accurate LRR classification can be impacted by registration performance, particularly with conformal radiotherapy’s steep dose gradients. To address this, this workflow utilises DIR due to its improved localisation of mapped rGTVs compared with other registration methods.^7^^,^^19^ DIR accuracy is evaluated using the TRE of site-specific ROIs, to determine DIR accuracy in the local region of the LRR. As DIR accuracy could impact classification, one parameter for classification directly incorporates the TRE, by evaluating the dose to a sphere volume around the rGTV centroid with a radius equal to TRE. Additionally, while this workflow internally performs DIR and evaluates its accuracy, research teams and hospital sites may have independent local registration assessment protocols. To facilitate the workflow’s generalisability the user can input their own evaluation of DIR performance in-place of those built into the workflow.

The other parameter central to LRR classification is the rGTV centroid. This workflow uses a centroid-based method that asserts the mapped rGTV the centroid as the oncogenic foci of the recurrence,^7^^,^^8^^,^^11^^,^^20^ under the assumption of isotropic growth. While we recognise this assumption may not always hold, especially in large recurrence volumes or anatomical boundaries where additional visual inspection may be required, previous work indicates that prediction of isotropic expansion from central foci can serve as a suitable approximation.^21^^,^^22^

Finally, a site-independent classification workflow allows exploration of LRR patterns across different cancer or trial protocols. These classifications could directly inform clinical decision-making, such as adapting treatment strategies based on observed failure patterns. Central failures, Type A and C, are classified as central high-dose or elective-dose failures, respectively, where the recurrence centroid and sphere are entirely within the high or intermediate-risk regions. Of particular interest, Type A high-dose failures where patients have received maximal radiation dose could suggest inherent radioresistance and prompt further investigation into causal patient characteristics and alternative treatment strategies or therapies such as novel drug combinations. Peripheral failures, Types B and D, are failures on the borderline of high- or elective-dose regions, may warrant investigation into treatment parameters and dose delivery, such as target delineation and structure margins. Extraneous failures, Type E, are identified in low-dose regions. These classifications could indicate spread either prior to radiotherapy, or post-radiotherapy due to radioresistance or inadequate dose. The failures could have increased incidence for specific treatment protocols, necessitating careful analysis with treatment covariates and patient characteristics.

## Conclusion

This unified workflow for classifying LRR using the treatment planning dose distribution has been successfully implemented on two different cancer sites and patient cohorts. It accomodates variations in delineated structures, prescribed dosages, and treatment delivery, and lays the groundwork for failure pattern analysis that incorporates dose into LRR classifications. This method promotes standardised terminology for failure reporting, cross-centre validation, and sharing developments in best clinical practice for LRR review, bringing us one step closer to understanding LRR incidence across major malignancies.
